# The other side of the coin: oxytocin decreases the adherence to fairness norms

**DOI:** 10.3389/fnhum.2012.00193

**Published:** 2012-06-28

**Authors:** Sina Radke, Ellen R. A. de Bruijn

**Affiliations:** ^1^Donders Institute for Brain, Cognition and BehaviorRadboud University Nijmegen, Netherlands; ^2^Department of Clinical, Health and Neuropsychology, Leiden Institute for Brain and CognitionLeiden University, Netherlands

**Keywords:** oxytocin, fairness, generosity, ultimatum game, dictator game, social norms, prosocial behavior, social decision-making

## Abstract

Oxytocin (OXT) has been implicated in prosocial behaviors such as trust and generosity. Yet, these effects appear to strongly depend on characteristics of the situation and the people with whom we interact or make decisions. Norms and rules can facilitate and guide our actions, with fairness being a particularly salient and fundamental norm. The current study investigated the effects of intranasal OXT administration on fairness considerations in social decision-making in a double-blind, placebo-controlled within-subject design. After having received 24 IU of OXT or placebo (PLC), participants completed a one-shot Dictator Game (DG) and played the role of the responder in a modified version of the Ultimatum Game (UG), in which an unfair offer of eight coins for the proposer and two coins for the responder is paired with either a fair-(5:5) or no-alternative (8:2). Rejection rates were higher when a fair alternative had been available than when there was no alternative to an unfair offer. Importantly, OXT did not de-or increase rejection rates overall, but reduced the sensitivity to contextual fairness, i.e., the context of alternatives in which an offer was made. As dictators, participants allocated less coins to the recipient when given OXT than when given PLC, indicating a decline in generosity. These results suggest that OXT decreases the adherence to fairness norms in social settings where others are likely to be perceived as not belonging to one's ingroup. While our findings do not support the prosocial conception of OXT, they corroborate recent ideas that the effects of OXT are more nuanced than assumed in the past.

## Introduction

The neuropeptide oxytocin (OXT) has received much attention for its role in social cognition and prosocial behavior (Meyer-Lindenberg, 2008; Macdonald and Macdonald, 2010). Previous studies have revealed that OXT strengthens cooperation by stimulating trust (Kosfeld et al., 2005; Baumgartner et al., 2008; Delgado, 2008; Mikolajczak et al., 2010a,b), generosity (Zak et al., 2007), and social perception (Guastella et al., 2008a,b; Keri and Benedek, 2009; Gamer et al., 2010), suggesting a strong association between OXT and empathy (Zak et al., 2007; Barraza and Zak, 2009).

However, recent evidence specifies that these effects are more nuanced than once assumed and often moderated by situational or personal characteristics (Bartz et al., 2011). Some findings even point to rather “antisocial” effects of OXT (Bartz et al., 2011), such as increased envy and Schadenfreude (Shamay-Tsoory et al., 2009) as well as ingroup-favoritism and aggression towards outgroup members (De Dreu et al., 2010, 2011a). Similarly, OXT diminishes cooperation when social information about the interaction partner is lacking (Declerck et al., 2010) and loses its trust-enhancing effect when interaction partners are perceived as unreliable (Mikolajczak et al., 2010a).

Since the central decisions in our life occur during interactions with others, commonly shared beliefs, i.e., social norms, provide a useful framework for our decisions and deeds. Fairness is a very elementary and salient norm, for which a preference is already observable in young children (Takagishi et al., 2010; Blake and Mcauliffe, 2011). These social preferences are frequently investigated with one-shot games, among others, the Ultimatum Game (UG, Güth et al., 1982) and the Dictator Game (DG) (Forsythe et al., 1994; Fehr and Camerer, 2007; Fehr, 2008). Both games involve monetary allocations between two players, with the first player offering a division. In the UG, the second player can decide whether to accept or reject this proposal. If accepted, the stake is split as proposed. If the offer is rejected, neither player receives anything. In the DG, on the contrary, the decision in unilateral on behalf of the allocator and the second player must accept any offer, thus remaining utterly powerless. In both games, empirical data differs from a “rational” approach of maximizing one's payoff (Güth et al., 1982).

The study by Zak et al. (2007) is, up to now, the only one to investigate the influence of OXT on the behavior in the UG and the DG. Here, participants were asked to indicate the value they would choose if they were assigned to be proposers (offer), responders (minimum acceptable offer or, in other words, rejection threshold) and dictators (endowment/giving), respectively. OXT enlarged the (positive) difference between proposers' offers and their rejection threshold in the UG, while leaving rejection thresholds and DG giving unchanged. The authors conclude that OXT increases generosity, based on the definition that generosity means giving away more than the recipient needs or expects. In fact, in this study, proposers were not informed about the actual expectations (or needs) of the second player, but made hypothetical “what-if”-decisions before being assigned to a role. Zak et al. (2007) propose that this procedure, in combination with OXT, stimulates perspective-taking and empathy in the UG, and in turn motivates to reduce the negative emotional reaction of the other player. They do not, however, provide an explanation why this only holds in the role of proposers and not responders. A true concern for others' welfare should also be evident in altered rejection thresholds and DG allocations. An OXT-induced “generosity” that is only evident when the second player has the power of rejecting one's offer, which would leave oneself empty-handed, does not seem very generous after all, but might reflect strategic considerations (see also De Dreu, 2012). In line with the conclusions of Zak et al. (2007), no OXT effects on the decision to donate have been found (Barraza et al., 2011). A different study by the same authors, however, reported increased generosity in unilateral monetary allocations in relation to OXT levels in blood (Barraza and Zak, 2009). With respect to the relation between genetic variations in the OXT receptor and monetary transfers, results are similarly divergent (Israel et al., 2009; Apicella et al., 2011). Fehr (2008) and Conlisk (2011) even reason that OXT does not boost generosity or prosociality, which is also supported by the absence of OXT effects on the back-transfer of trustees in a trust game (Kosfeld et al., 2005). Likewise, the initial transfer of investors did not differ between OXT and placebo (PLC) (Baumgartner et al., 2008) or when trustees were depicted as unreliable (Mikolajczak et al., 2010a). All in all, the experimental findings are mixed and it remains thus unresolved whether OXT actually motivates prosociality by stimulating perspective-taking.

A modified version of the UG developed by Falk et al. (2003) allows for a more thorough examination of perspective-taking particularly from the side of responders. Here, the proposer chooses from a fixed set of two distributions of the stake. An unfair offer of eight coins for the proposer and two coins for the responder is paired with different alternatives, most critically either a fair-(5:5) or no-alternative (8:2). Previous studies using the modified UG paradigm have repeatedly demonstrated that rejection rates are higher when there was a fair-alternative than when there was no-alternative to an unfair offer (Falk et al., 2003; Sutter, 2007; Güroğlu et al., 2009; Radke et al., 2012). Although identical in terms of absolute payoff, the unfair offers differ with respect to signaling fairness depending on the available alternative. Importantly, pairing an unfair offer (8:2) with a fair alternative (5:5) signifies an explicit violation of fairness norms because the proposer clearly preferred *not* to offer an equal split, but favored an unfair division (Radke et al., 2012). Incorporating proposers' perspective and judging this behavior as unkind and unfair underlies the increased tendency to reject. In contrast, when no alternative was available, rejection is solely based on disliking the unfair outcome as such, i.e., inequity aversion (Falk et al., 2003). Developmental studies support the notion that the sensitivity to this manipulation of “context”, i.e., the alternative offer (as in Güroğlu et al., 2009; Radke et al., 2012), reflects perspective-taking (Sutter, 2007; Güroğlu et al., 2009).

We used the modified version of the UG to contrast behavior in response to unfair offers when no alternative was available to unfair offers which were deliberately chosen over a fair alternative, i.e., an equal split. Here, the no-alternative condition captures the tendency to dislike and reject unequal outcomes, i.e., inequity aversion, which is a basic social preference (Radke et al., 2012). In accordance with previous findings (Falk et al., 2003; Sutter, 2007; Güroğlu et al., 2009; Radke et al., 2012), we expected responders' rejection rates to remain substantial, but lower than in the fair-alternative condition. The difference in rejection rates between these two conditions assesses how sensitive responders are to the alternative, but unselected, offer that had initially been available to proposers. In other words, the sensitivity to the context in which an unfair offer occurred goes beyond pure inequity aversion by stirring social expectations about fairness. Importantly, examining responder behavior in an UG setting allows for distinguishing social norm concerns from other motivational dynamics that accompany proposals, e.g., the strategic rationale of offering fair splits to minimize rejection and thereby maximize self-gain. The current study is the first to assess the role of OXT on actual responder *behavior* in the UG, i.e., reactions to others' proposals. If OXT promotes prosociality and perspective-taking in general, then a larger sensitivity to context should emerge. In a similar vein, unilateral “prosocial” allocations should be higher after OXT administration. The DG has been highlighted as a measure of unconditional prosociality and altruism (Camerer and Thaler, 1995; Conlisk, 2011). On the other hand, however, newer research suggests (De Dreu, 2012) that OXT motivates only parochial cooperation. When others are unknown or unfamiliar, OXT can effectively reduce cooperative conduct (Declerck et al., 2010). Since no personal inferences about the other players could be drawn in the current setting, they are likely to be perceived as not belonging to the same group, i.e., ingroup, as oneself. Moreover, the UG involves a limited stake, i.e., coins, with the payoffs for the two players being inversely related. Particularly when competing for the same resources, potential prosocial tendencies or privileges might not extend to principally unknown interaction partners. Consequently, we expected participants to adhere less to social norms of reciprocity and fairness when distributing money with an anonymous other. Still, as the results from previous studies are mixed, the character of the current experiment remains rather explorative.

## Materials and methods

### Participants

Twenty-four male volunteers (*M* age = 21.46, SD = 1.93 years) participated in this study. All of them were students and recruited through advertisements placed across campus.

All participants were healthy and did neither report current nor a history of neurological or endocrine disease, medication, and drug or alcohol abuse. Exclusion criteria included age of <18 or >30, smoking more than five cigarettes per day, participation in another pharmacological study or blood donation within the last two months, and suffering from fever, common cold or allergic rhinitis (“hay fever”) on the day of testing. Participants were asked to abstain from caffeine, alcohol and nicotine for 24 h as well as from eating and drinking (except water) 2 h prior to substance administration.

All participants gave written informed consent to the procedures which were in accordance with the Declaration of Helsinki and had previously been approved by the Medical Ethics Committee of the Radboud University Nijmegen Medical Center (Commissie Mensengebonden Onderzoek Region Arnhem-Nijmegen). Participants were paid for participation.

### Pharmacological procedure

A randomized, placebo-controlled, double-blind within-subjects design was used in this study. Participants received OXT (Syntocinon; Novartis) or a saline solution via a nasal spray during two sessions separated by 14 days. All sessions were scheduled for weekdays, started at 10 a.m. and involved two participants, who did not know each other before, being tested simultaneously. In order to avoid any bias due to potential differences in scent between the OXT and the saline spray, the experimenter was not present during substance administration. An independent assistant who was blind to the experimental hypotheses supervised the procedure and left immediately after substance administration. Participants self-administered the nasal spray with three puffs per nostril (each with 4 IU OXT, i.e., a total dose of 24 IU). To control for belief effects, participants as well as the experimenter had to indicate at the end of each session which substance they think was administered. In addition, mood questionnaires were completed throughout the sessions to assess nonspecific effects of OXT. Several tasks were carried out after a waiting period of approximately 40 min, a time window derived from earlier OXT and related peptide nasal spray studies (Born et al., 2002; Kosfeld et al., 2005; Domes et al., 2007; Gamer and Büchel, 2012), with subjects starting the UG and DG approximately 75 min after substance administration. Participants were not allowed to talk to each other during the UG and DG.

### Materials

#### Modified ultimatum game

**Figure 1:**
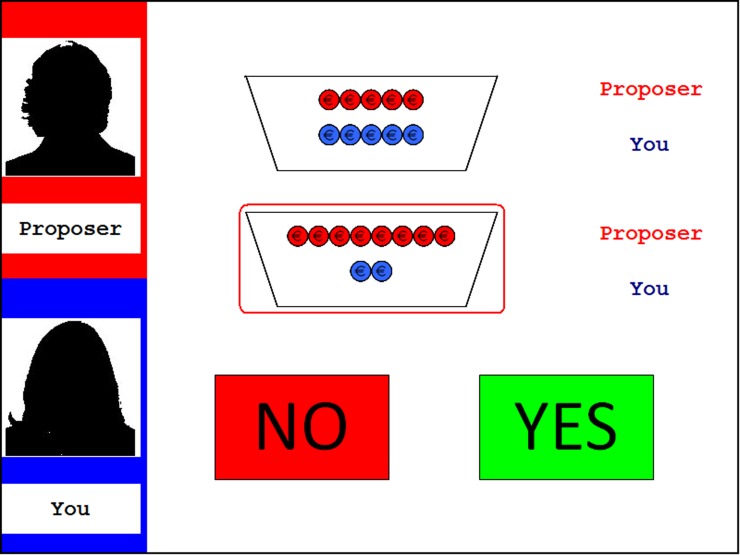
**Display of the decision phase in the fair-alternative condition of the modified UG.** The left panel shows the name and silhouette of the proposer at the top (here “Proposer”) as well as the name of the participant underneath (here “You”). The two potential distributions are specified by red and blue coins (red for the proposer, blue for the responder; here 8:2 vs. 5:5). The selected offer is encircled in red. The participant has to decide whether to accept (“Yes”) or reject (“No”) the offer via button press.

***Procedure.*** Participants played the role of the responder in a computerized version of the modified UG. Each trial started with a fixation cross (1000 ms), followed by the presentation of the two available options (1000 ms). Next, the selected offer was encircled in red (1000 ms). Subsequently, “Yes” and “No” icons were presented while the alternatives remained visible (as depicted in Figure 1). The task being self-paced, participants had unlimited amount of time to press one of two buttons on the keyboard to indicate their decision. Participants' response remained on the screen for 2000 ms before the next round started.

Participants were led to believe that they were coupled with data from subjects who had previously participated as proposers. They were told that they would play every round with a new partner who would make an offer by selecting one of the two options and their task was to decide whether to accept or reject that particular offer. If accepted, the coins were distributed as proposed; if rejected, neither player received anything. Participants were notified that at the end of the experiment, a random number of rounds would be selected to determine their payoff and that proposers would be paid in the same manner after all data from responders had been collected. It was pointed out that participants' decisions affected both their own and the other players' financial outcome. It was ensured that participants' earnings varied between the two experimental sessions and between participants sitting in the same room. None of the participants indicated doubt about the cover story or about the bonus not being linked to their actual choices.

***Design and analyses.*** In order to contrast behavior in response to unfair offers (8:2) when no alternative (8:2 vs. 8:2) was available to unfair offers which were deliberately chosen over a fair alternative (5:5 vs. 8:2), i.e., an equal split, a repeated measures ANOVA was conducted for the rejection rate of unfair offers with substance (two levels: OXT vs. PLC) and context (two levels: fair vs. no alternative) as within-subject factors. Hence, the factor context pertains to the alternative outcome that had not been selected. The fair-alternative condition can be seen as an explicit version of the classic UG where any offer is usually compared to a potential equal split. Pairing an unfair offer with a fair alternative consistently leads to highest rejection rates (Falk et al., 2003; Sutter, 2007; Güroğlu et al., 2009; Radke et al., 2012). In contrast, the rejection rate in the no-alternative condition is likely to reflect the basic tendency for inequity aversion (Falk et al., 2003; Ohmura and Yamagishi, 2005). Although the two identical distributions do not permit a real choice for proposers, responders' rejection rates remain substantial (Falk et al., 2003; Sutter, 2007; Güroğlu et al., 2009; Radke et al., 2012). Importantly, the difference in rejection rates between the no-alternative and the fair-alternative condition can be regarded as a measure of the sensitivity to contextual fairness.

Two additional distributions were used as to induce variance in the set of offers and to avoid suspicion from participants being faced with only 8:2 and 5:5 splits on all trials. For this purpose, we included hyperfair (2:8 vs. 8:2) and hyperunfair (10:0 vs. 8:2) conditions in the game. However, for the hyperfair condition, it is still unresolved what motivates the decision to accept or reject (Güroğlu et al., 2009; Sutter, 2007). Importantly, with regard to fairness norms, both offers are equally unfair, one being advantageous to the proposer and the other being advantageous to the responder. As it is not obvious which choice is favorable according to social norms and expectations, interpreting this condition remains particularly challenging. With regard to the hyperunfair condition, results based on similar paradigms are mixed. Whereas Falk et al. (2003), Güroğlu et al. (2009) and Radke et al. (2012) do not find significant differences between the hyperunfair and no-alternative condition, the experiment of Sutter (2007) reveals higher rejection rates in the no-alternative (8:2) than in the hyperunfair-alternative condition (10:0) for university students. These inconsistent findings warrant caution when interpreting the results from the hyperunfair-alternative condition and have entailed its exclusion from the design and analyses previously (Güroğlu et al., 2010).^1^ For these reasons, we restricted the analyses to the two levels of context that permit a solid, unambiguous investigation of the role of OXT in fairness considerations.

Each combination of selected and unselected offers was presented 16 times (counterbalanced for proposers' gender and position of the unfair offer). As the no-alternative condition leads to an 8:2 offer for either alternative, an unfair offer (8:2) was presented in five of the eight conditions, equivalent to 80 trials. The three genuine alternative offers (i.e., 5:5, 2:8 or 10:0) were selected on 48 trials, yielding 128 trials in total. Contrary to subjects' belief, all choices were computer-generated.

#### Dictator game

After completion of the modified UG, participants played a single-trial DG with an anonymous other who was represented by a gender-ambiguous silhouette and name. Ten red coins were presented similar to the display in the modified UG. Participants had an unlimited amount of time to choose how many coins they wanted to give to the other player who, as it was emphasized, could not influence the outcome, but would be paid contingent upon their decision. Responses were made by pressing the corresponding number on the keyboard.

## Results

### Modified UG

**Figure 2:**
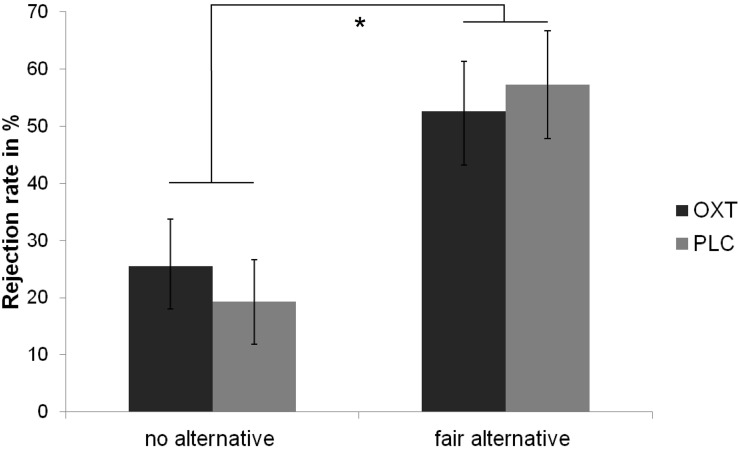
**Rejection rates of unfair offers with regard to the alternative offers and the substance received.** Overall mean percentage and standard errors of rejection of 8:2-offers are displayed. The main effect of context is indicated by an asterisk(^*^), *p* < 0.01.

The ANOVA revealed a main effect of context, *F*_(1, 23)_ = 15.80, *p* < 0.01, η^2^ = 0.41, indicating that rejection rates were higher in the fair-alternative condition (*M* = 54.95%) than in the no-alternative condition (*M* = 22.4%). Moreover, there was an interaction between substance and context, *F*_(1, 23)_ = 4.44, *p* < 0.05, η^2^ = 0.16. Further analyses demonstrated that the difference in rejection rates between the fair-alternative condition and the no-alternative condition was smaller after OXT administration (*M* = 27.08) than after PLC (*M* = 38.02). The effect of substance was not significant, *F*_(1, 23)_ = 0.02, *p* = 0.88, η^2^ < 0.01. Rejection rates are depicted in Figure 2.

### Dictator game

**Figure 3:**
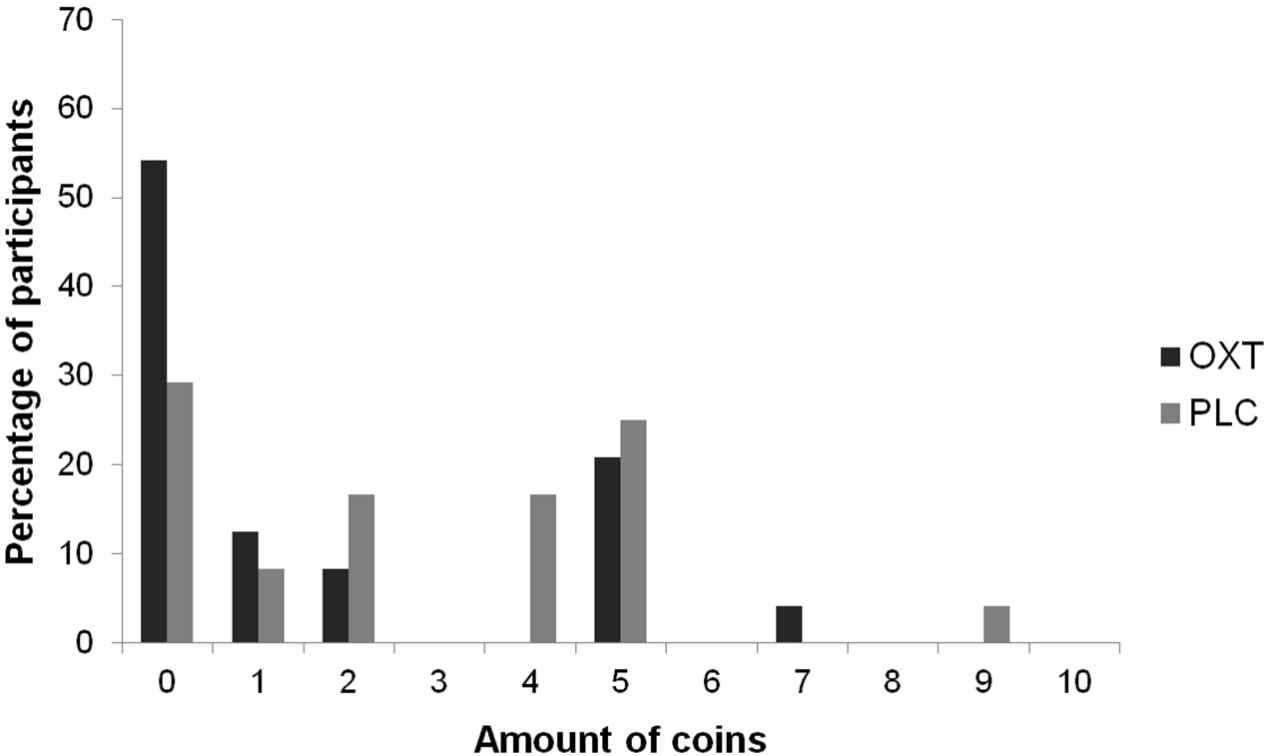
**Distribution of Dictator allocations after OXT vs. PLC administration**.

The number of coins allocated to the recipient was smaller when participants had received OXT (*M* = 1.63, *SD* = 2.3; *Median* = 0) than when they had received PLC (*M* = 2.71, *SD* = 2.44; *Median* = 2), *Z* = −2.06, *p* = 0.04 (two-tailed Wilcoxon Test). Figure 3 depicts the histogram of allocations. In the PLC condition, the distribution is bimodal, with seven participants giving zero coins (29.2%) and six giving five coins, i.e., half of the stake (25%). After OXT administration, the distribution of endowments is unimodal, peaking at zero (*N* = 13; 54.2%) and five participants splitting equally (20.8%).

### Effects of order or participants' belief of substance administration

Adding the order of substance administration or subjects' belief about the substance administered as between-subject factors to the ANOVAs did not yield any significant effects or interactions (all *p*s > 0.28). Neither participants nor the experimenter were able to detect the correct order of substance administration above chance level (participants: *M* = 47.83%; *t*(22) = −0.204, *p* = 0.84; experimenter: *M* = 33.13%; *t*(23) = −1.696, *p* = 0.10).

## Discussion

This study aimed to explore the role of OXT in fairness considerations that imply social norms. It was the first experimental approach of administering OXT intranasally in order to assess actual responder *behavior* in an UG setting. The modified version of the UG allowed for investigating perspective-taking from the side of responders as this role is related less to strategic, but more to fairness considerations. Additionally, for a direct comparison with the only previous pharmacological study using the UG/DG (Zak et al., 2007), the DG was included to capture unconditional generosity.

Rejection rates in the modified UG were higher when a fair alternative had been available than when there was no alternative to an unfair offer-an effect that has been frequently shown (Falk et al., 2003; Sutter, 2007; Güroğlu et al., 2009; Radke et al., 2012). Importantly, OXT did not generally de-or increase rejection rates, but reduced the sensitivity to contextual fairness. Whereas a typical, bimodal distribution of allocations was observed in the PLC condition of the DG, OXT skewed this pattern in the direction of enlarging one's own gain. Taken together, OXT appears to decrease the amount to which one acts according to social rules and norms. In the DG, it decreases unconditional generosity, and in the UG, the alternative, unselected offer is taken less into account. Notably, participants were less responsive to cues that stimulate perspective-taking by means of inferring proposers' motives for selecting an unfair offer (e.g., Güroğlu et al., 2009).

These results are clearly at odds with the notion of OXT inducing generally prosocial tendencies (Zak et al., 2007; Meyer-Lindenberg, 2008; Macdonald and Macdonald, 2010). Instead, they fit with recent evidence suggesting rather “antisocial” effects of OXT (Bartz et al., 2011), ranging from negative interpersonal feelings, such as increased envy and Schadenfreude (Shamay-Tsoory et al., 2009) to intergroup behavior, e.g., ingroup-favoritism (De Dreu et al., 2010, 2011a). Importantly, in the absence of social information about the interaction partner, OXT decreases cooperation (Declerck et al., 2010). Along these lines, De Dreu (2011b, 2012) argues that OXT-induced “goodwill” is not general, but in fact parochial, and does not extend to members that are perceived to be unreliable (Mikolajczak et al., 2010a) or do not belong to one's ingroup. This limited benevolence is likely to sustain intra-group reciprocity and fits with findings from animal literature (e.g., Campbell, 2008).

Social norms are not merely shared by others, but, importantly, also sustained by others' endorsement and therefore serve the cohesion of social groups. Violating social expectations often leads to disapproval by others and, depending on the nature of the particular norm, feelings of anxiety, guilt or embarrassment on the side of the violator (Elster, 1989). Importantly, these negative emotions can also arise when anticipating to violate social norms (Elster, 1989). Enhanced amygdala activation has been associated with own intentional norm violations (Berthoz et al., 2006) as well as with judging actions as reflecting deceptive intentions (Grèzes et al., 2004). Even in anonymous settings, individuals avoid circumstances that enable them to deceive others to their own financial advantage (Shalvi et al., 2011). Rooted in the desire not to behave in an immoral and socially inconsiderate manner, people are inclined to satisfy others' expectations and to avoid social interactions that involve conflicting interests or a temptation to exploit (Dana et al., 2006; Shalvi et al., 2011). Given that OXT attenuates responses to stress, threat and anxiety, particularly in social situations (Heinrichs et al., 2006; Heinrichs and Domes, 2008; Ditzen et al., 2009; Norman et al., 2010; Bartz et al., 2011), OXT is likely to diminish the concern about other people's disapproval. In patients with social anxiety disorder, OXT reduced exaggerated negative mental self representations (Guastella et al., 2009). Therefore, acting against the rules of social conduct could be viewed as less threatening and more permissive, resulting in being a more feasible behavioral option.

Apart from its anxiolytic effects, OXT is involved in facilitating social categorization (De Dreu, 2012). Although we did not intend to manipulate group membership, the setting of our experiment may have contributed to such a classification. The other players with whom participants interacted via the computer were represented by black silhouettes and names consisting of their first name and the first letter of their last name. Moreover, every UG round was played with a new partner, preventing participants from familiarizing with them and developing reciprocal patterns. In contrast, a fellow participant of the same gender was present in the same room and busy with the same task. This might have induced a distinction between the fellow participants being similar to oneself and belonging to the same group, whereas the other players changed frequently and did not share these “established” commonality of the ingroup. OXT might have fostered the perception of this contrast, which is in line with previous evidence on unkind behavior towards non-ingroup members (De Dreu, 2012). Bearing in mind that the gender-ambiguous silhouette and name used in the DG does not allow for deducing any identity-not even for a fundamental inference based on gender-it appears that “antisocial” effects of OXT are inversely related to the information available about the other player (see also Declerck et al., 2010). However, these speculations need to be directly tested in future studies since our design did not manipulate intergroup dynamics on purpose. In addition, it should be investigated in how far OXT might alter the perception of and reaction to ambiguous social cues.

Note that our study differs from the one of Zak et al. (2007) in two central methodological aspects: First, participants of Zak et al. made choices in rather hypothetical situations, i.e., as if they were proposers, responders, and dictators, preceding the assignment of definite roles. In contrast, in the current study, participants (as responders) always reacted to offers from proposers, which puts more emphasis on actual decision behavior. Closely related is the lack of an explicit reference point in the classic UG (as used by Zak et al.) so that the fairness norm of a potential equal split remains implicit (Radke et al., 2012). By pairing an unfair offer with a fair alternative (as in the current design), an explicit violation of fairness norms can be signified and context effects can be captured. Second, Zak et al. (2007) administered 40 IU in a between-subject manner, whereas the current study made use of a dose of 24 IU and a within-subjects design. Although 24 IU has emerged as the conventional dosage for OXT research, the effects of dose, e.g., whether they are linear or follow a different functional mapping, should be thoroughly investigated in clinical trials. In the absence of such trials, the exact pharmacokinetics of OXT remain unknown. Importantly, however, the current study is based on data suggesting a time window of up to 100–120 minutes in CSF after intranasal neuropeptide administration (Born et al., 2002) and OXT effects for at least 90 min (e.g., Domes et al., 2010; Gamer et al., 2010; Gamer and Büchel, 2012). Recently, results were reported for tasks starting 75–85 minutes after OXT administration, with the entire experimental session lasting from 45 until 120 minutes post-administration (Ellenbogen et al., 2012).

As the DG was always administered after the UG, we cannot entirely rule out possible carry-over effects from the previous interactions in which participants faced many unfair offers. Yet, it seems unlikely that the task order is responsible for the current results as the effect was restricted to the OXT session and not present when participants received PLC. Besides, we found no effects of session order or mood that might explain our results in terms of unspecific substance effects.

In conclusion, our results indicate that OXT reduces the sensitivity to fairness considerations based on perspective-taking (UG) and generosity (DG). The current findings add to a growing body of literature on differential effects of OXT that essentially depend on situational or personal characteristics (Bartz et al., 2011) as well as the nature of social cues (Declerck et al., 2010; De Dreu, 2012). Tuning one's behavior according to the attributes of one's interaction partner is highly adaptive and restricting prosocial behavior to one's ingroup is likely to strengthen group cohesion and fitness. A facilitated social categorization, e.g., based on group membership, can be useful under conditions of uncertainty as it reduces the threat of non-reciprocation. Along these lines, the currently demonstrated decreased adherence to social norms is usually only advantageous in the short run and towards non-ingroup members. Therefore, replications and extensions to long-lasting social relationships are necessary to investigate the mechanisms behind OXT-induced alterations of social behavior and their modulation by situational and interpersonal factors. After all, it might be beneficial that OXT does not motivate prosocial tendencies towards anyone.

### Conflict of interest statement

The authors declare that the research was conducted in the absence of any commercial or financial relationships that could be construed as a potential conflict of interest.

## Footnotes

Note that there was another condition in which proposers were deprived of their control over an offer, which, however, extends the focus of the current paper and will be reported elsewhere.
